# Novel Anticoagulants for Stroke Prevention in Atrial Fibrillation: A Comprehensive Review

**DOI:** 10.7759/cureus.65347

**Published:** 2024-07-25

**Authors:** Prinka Perswani, Ritesh Croos Yogarajah, Mohammed Saifuddin, Alisha Lakhani, Jagruti Dasi, Vanshika Bhardwaj, Bhavana Kumar, Ojasvi Raina, Nicole Fletcher, Grace Jomy, Pracruti Iyer, Jai Pasi, Kanika Tayal, Hasim Reza

**Affiliations:** 1 Internal Medicine, Liaquat University of Medical and Health Sciences, Jamshoro, PAK; 2 Cardiology, Jonelta Foundation School of Medicine, University of Perpetual Help System DALTA, Las Piñas, PHL; 3 Medicine, Navodaya Medical College, Raichur, IND; 4 Research, Research MD, Vadodara, IND; 5 Medicine, Shantabaa Medical College, Amreli, IND; 6 Medicine, Lokmanya Tilak Municipal General Hospital, Mumbai, IND; 7 Cardiology, Lala Lajpat Rai Memorial Medical College, Meerut, IND; 8 Medicine, Jagadguru Jayadeva Murugarajendra Medical College, Davanagere, IND; 9 Cardiology, Avalon University, Willemstad, CUW; 10 Medicine, Dr. Somervell Memorial CSI Medical College & Hospital, Karakonam, IND; 11 Medicine, Nil Ratan Sircar Medical College and Hospital, Kolkata, IND; 12 Medicine, BKL Walawalkar Rural Medical College, Sawarde, IND; 13 Medicine, Dr. Ram Manohar Lohia Institute of Medical Sciences, Lucknow, IND; 14 Medicine, Central America Health Sciences University, Ladyville, BLZ

**Keywords:** anticoagulants, vitamin k antagonist anticoagulant, direct oral anticoagulant (doac), newer oral anticoagulants, atrial fibrillation (af), stroke

## Abstract

Atrial fibrillation (AF) is a prevalent cardiac arrhythmia associated with an increased risk of stroke due to disrupted heart function and potential clot formation. This review examines current management strategies for stroke prevention in AF, focusing on the efficacy, safety, and long-term outcomes of anticoagulation therapies. Anticoagulants, including novel oral anticoagulants (NOACs) and vitamin K antagonists, play a crucial role in reducing stroke risk by preventing clot formation in the heart.

Recent studies highlight NOACs as superior alternatives to traditional therapies, offering improved safety profiles and enhanced patient adherence. Despite the risk of bleeding complications, judicious use of anticoagulants significantly improves clinical outcomes in AF patients.

The review synthesizes evidence from clinical trials and meta-analyses to underscore the pivotal role of NOACs in transforming stroke prevention strategies in AF. Moreover, it discusses emerging interventions such as left atrial appendage occlusion and emphasizes the importance of personalized, patient-centered care in optimizing treatment decisions for AF patients at risk of stroke.

## Introduction and background

Atrial fibrillation (AF), characterized by irregularly irregular and rapid heart rate, stands as one of the most prevalent cardiac arrhythmias seen in clinical practice across the world. It accounts for about 35% of hospital admissions from cardiac rhythm disorders [1]. In AF, the disruption in the heart’s electrical activity compromises its ability to effectively pump blood. Consequently, reduced blood flow through the upper chambers can increase the risk of blood clot formation within the heart. These thrombi can potentially embolize and reach the cerebral vasculature via the systemic circulation, resulting in a cerebrovascular accident. A stroke occurs when the blood supply to a part of the brain is interrupted or reduced, depriving brain tissue of oxygen and nutrients. Ischemic stroke, the most common type, results from a blockage or clot within a blood vessel supplying the brain, often originating from blood clots formed elsewhere in the body [2].

The relationship between AF and stroke is well-established, with AF significantly increasing the risk of stroke by four to five times. Around 15-20% of ischemic strokes are attributed to AF, emphasizing the impact of this tachyarrhythmia on the incidence of stroke. Moreover, strokes associated with AF tend to be more severe and have a higher mortality rate in comparison to strokes unrelated to the cardiac condition. Data suggest that AF confers a five-fold increase in stroke risk and a two-fold increase in mortality. These figures emphasize the importance of stroke prevention in AF [2,3].

A comprehensive multi-dimensional approach comprising risk assessments, such as CHA2DS2-Vasc score and ORBIT (Outcomes Registry for Better Informed Treatment of Atrial Fibrillation) score, appropriate intervention, patient education, and regular follow-ups help optimize living standards for AF patients. Effective anticoagulation strategies have resulted in a 65% reduction in mortality due to stroke in AF patients [4].

According to the 2020 European Society of Cardiology, the pathway for AF management includes the following: A - avoid stroke, B - better symptom-based treatment, C - cardiovascular and other comorbid risk reduction [5]. Anticoagulants are the mainstay of treatment to prevent strokes. At least 24 hours of ECG monitoring after stroke is recommended to rule out AF as it is the leading cause and risk can be reduced by the use of oral anticoagulants, especially direct-acting oral anticoagulants (DOACs) rather than the use of vitamin K-dependent oral anticoagulants [6].

Anticoagulants play a pivotal role in stroke prevention among patients with AF. Often referred to as "blood thinners", these medications reduce the risk of stroke by preventing the formation of blood clots within the heart. Despite the inherent risk of bleeding complications associated with anticoagulant therapy, their judicious use can ultimately improve patient outcomes in AF. Anticoagulants, including vitamin K antagonists (VKAs) and novel oral anticoagulants (NOACs), represent cornerstone therapies for stroke prevention in AF. The initiation of anticoagulant therapy requires careful consideration of individual risk factors and benefits by healthcare providers to minimize the adverse effects [3].

The advent of DOACs has also been pivotal in transforming the clinical outcomes in preventing thromboembolic stroke in patients living with AF. An integrated and holistic approach, like the ABC pathway, has been shown to improve clinical outcomes such as a decrease in all-cause mortality and reduced risk of stroke [7].

Objective

This study aims to review the paradigm shift in the management strategies for the prevention of stroke in the existing literature. Additionally, the review evaluates the efficacy, safety, patient adherence, and long-term outcomes of various anticoagulation therapies for stroke prevention in AF. The increasing incidence of AF makes it essential to keep pace with recent advances and newer management strategies. Additionally, the review evaluates the impact of anticoagulation therapy on long-term clinical outcomes such as stroke recurrence, mortality rates, cardiovascular events, and quality of life measures. Finally, investigating patient adherence patterns aids in identifying barriers and facilitators to long-term treatment success, thus providing insights to guide evidence-based decision-making and optimize patient-centered care for individuals with AF at risk of stroke.

## Review

Historical perspective

Effective stroke prevention for patients with AF mostly requires the use of an oral anticoagulant, whether a VKA or a direct oral anticoagulant. Over the years, there has been a shift in the approach to treating strokes associated with AF, driven by the need for more effective anticoagulation therapies that can provide better patient outcomes in the long term. The following historical outline aims to give an overview of the evolution of treatment strategies over the recent decades (Table 1).

**Table 1:** Historical outline of treatment strategies over a decade.

Timeline	Treatment
Prior to 1950s	No specific anticoagulant therapies.
1950-1970	The first major milestone in anticoagulant therapy for atrial fibrillation came with the introduction of warfarin in the 1950s. Warfarin was discovered to have anticoagulant properties by decreasing the availability of active vitamin K leading to a reduction in blood coagulation [8].
1980-1990	In 1985, cardiologists and neurologists from different institutions collaborated on the design of a study called the Stroke Prevention in Atrial Fibrillation (SPAF) to evaluate the efficacy and safety of warfarin and aspirin, as individual treatments, in comparison to placebo for the prevention of ischemic stroke and systemic embolism. Through a randomized clinical trial, it was concluded that both warfarin and aspirin substantially reduce the risk of stroke and embolism in patients with non-valvular atrial fibrillation [9].
In the early 2000s	A significant shift in anticoagulation therapy with the introduction of direct oral anticoagulants (DOACs) [2].
2010-2015	The first DOAC to receive FDA approval in 2010 for stroke prevention in atrial fibrillation was dabigatran, a direct thrombin inhibitor [2].
2015 to Present	The introduction of the non-vitamin K antagonist oral anticoagulants (NOACs, also called direct oral anticoagulants, DOACs) has changed the approach to stroke prevention in atrial fibrillation (AF), such that the standard practice now is to recommend oral anticoagulation for stroke prevention unless the patient is at low risk. A new type of blood thinning medication called the direct inhibitors of factor XIa like asundexian and milvexian is currently being tested in phase III trials to prevent blood clots in various conditions, including stroke prevention in AF. These newer oral anticoagulant agents are expected to work better at preventing excessive bleeding while being as effective and safer compared to the current standard of AF treatment, which is DOAC [10].

Pathophysiology of stroke in atrial fibrillation

The most debilitating complication of AF is stroke. The traditional explanation of embolic stroke in patients with AF posits that the irregular beating of the atrium leads to inefficient contractions resulting in blood stasis that promotes the development of clots and their subsequent release into the circulation toward the brain [11].

The complex pathophysiology of thrombogenesis in AF involves multiple events. The pathogenic processes leading to thrombus formation within the left atrium (LA) and left atrial appendage (LAA) are most effectively explained through the framework provided by Virchow's triad [12].

**Figure 1:**
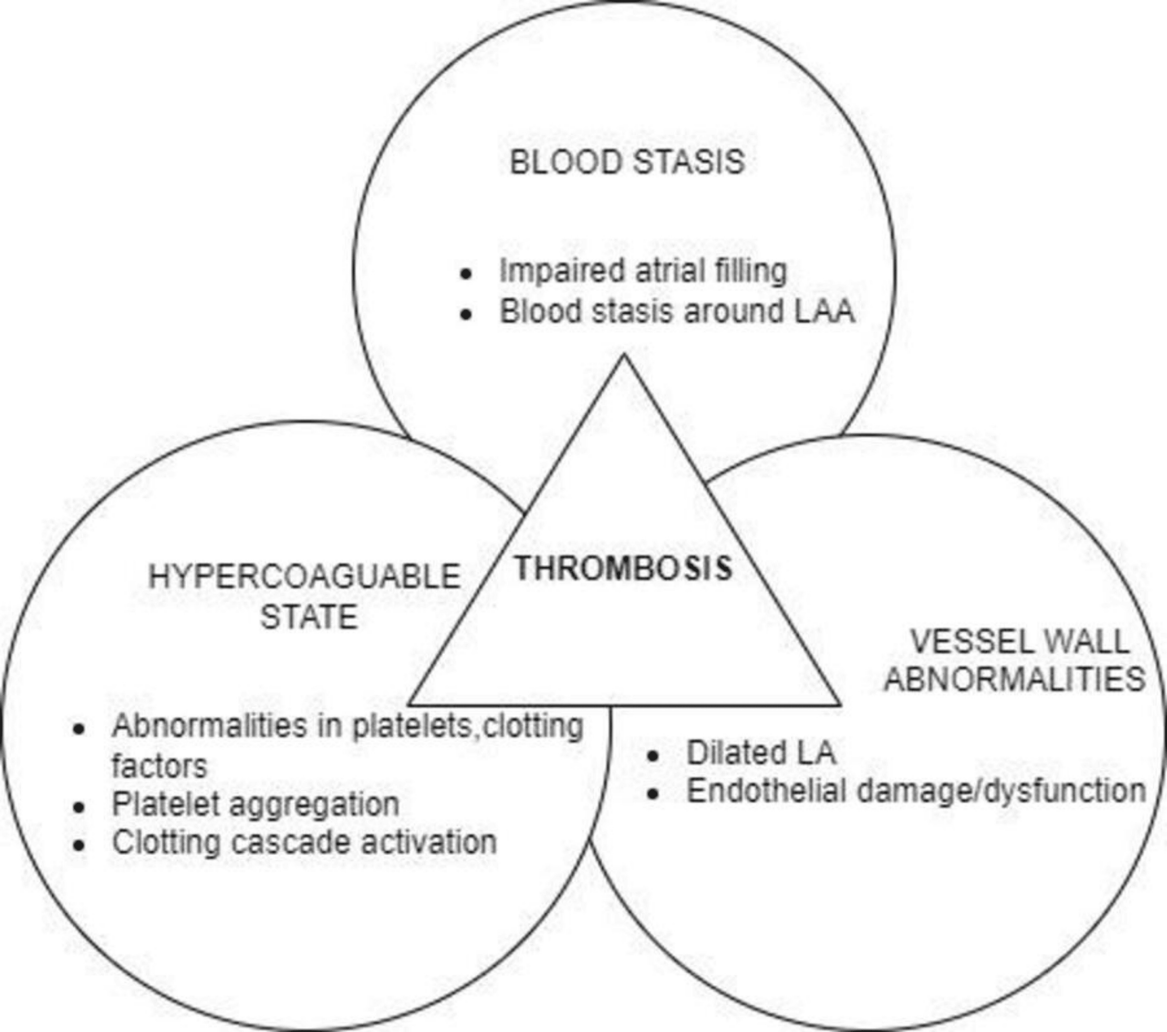
Virchow’s triad. LA: left atrium; LAA: left atrial appendage.

Virchow's triad includes abnormal blood flow and stasis, vessel wall abnormalities (e.g., structural heart disease and endothelial damage/dysfunction), and hypercoagulability (e.g., clotting factors and abnormalities in platelets) (Figure 1) [13].

The abnormal blood circulation within the left atrium, characterized by chaotic electrical impulses and a lack of synchronized atrial contractions, coupled with endothelial dysfunction and other thrombosis-promoting factors, frequently leads to the development of thrombus in the LAA. This thrombus can become detached and may migrate predominantly toward the cerebral arterial vasculature and cause stroke [14].

Research findings indicate that over 90% of emboli associated with non-valvular atrial fibrillation (NVAF) are derived from the LAA. LAA is a trabeculated tubular structure. It is a remnant of embryonic development and significantly aids left ventricular diastolic filling by acting as a reservoir for blood, adapting to pressure and volume changes. In individuals afflicted with AF, the LAA manifests discernible morphological changes, including dilatation, elongation, and a diminution in the volume of pectinate muscles; these changes make the LAA more prone to emboli formation. The structural heart changes include atrial fibrosis, which is associated with AF and stroke [15].

Atria fails to contract properly, resulting in increased pressure, dilatation, and stretch causing blood stasis and thrombi formation. Increased atrial stretch results in increased production of atrial natriuretic peptide and decreased formation of vasopressin, resulting in hemoconcentration within the LA. In AF patients, the plasma levels of platelet‐derived β‐thromboglobulin and platelet factor 4 are elevated. There are also elevated levels of coagulation parameters, such as thrombin‐antithrombin III complex, D‐dimer, fibrinogen, prothrombin fragments 1 and 2, and fibrinopeptide A, depicting a hypercoagulable state [15,16].

Abnormalities in atrial wall motion and blood flow cause injury of the endothelium in the atria leading to enhanced platelet aggregation and adhesion of activated platelets to the endothelium in the affected areas. The occurrence of endothelial injury correlates with an increase in tissue factor activity, which subsequently exacerbates platelet activation. Activated platelets act as a catalyst for assembling plasma factors into the prothrombinase complex. This process leads to thrombin formation, subsequent fibrin generation, and ultimately, thrombus consolidation [2].

The main mechanism of thrombogenesis includes atrial remodeling in patients with AF exacerbating stasis, amplifying the likelihood of thromboembolic events. The detection of spontaneous echocardiographic contrast during echocardiographic imaging serves as an autonomous indicator for stroke risk in individuals with AF. Changes happening in the inner layer of the LAA in individuals with AF can potentially contribute to the development of thrombogenesis. Numerous molecular pathways, involving inflammation, growth factors, nitric oxide, and the renin-angiotensin-aldosterone system, may collectively foster a prothrombotic state in individuals diagnosed with AF [15].

In summary, the convergence of hypercoagulability, atrial cardiomyopathy-associated endothelial injury, and compromised blood flow within dilated atrial chambers, notably the LAA with impaired contractility, collectively underlie the pathological cascade leading to thrombus formation within the left atrium. This intricate process substantially elevates the propensity for systemic thromboembolism and subsequent stroke [16].

Yet, the pathophysiology of thrombogenesis that links AF and stroke is not fully understood and is an area of active research to identify new therapeutic targets that would help prevent stroke from AF [12].

Current anticoagulation landscape

Risk Assessment

According to the recent guidelines from the European Society of Cardiology (ESC) and the American College of Cardiology (ACC)/American Heart Association (AHA), individuals who have been diagnosed with AF are recommended to undergo an annual evaluation for risk of thromboembolic events, on the basis of the CHA2DS2-VASc score. This scoring system scrutinizes the common clinical risk factors for stroke: congestive heart failure, hypertension, age, diabetes mellitus, stroke, vascular disease, and sex category (female). The presence of each clinical risk factor warrants one point with the exception of age greater than or equal to 75 years and a history of stroke, which is two points each (Table 2) [17,18].

**Table 2:** Risk factors and definitions for CHA2DS2-VASc score. Reference [17].

Risk factors and definitions			Points awarded
C	Heart failure	The presence of signs and symptoms of either right (elevated central venous pressure, hepatomegaly, dependent edema) or left ventricular failure (exertional dyspnea, cough, fatigue, orthopnea, paroxysmal nocturnal dyspnea, cardiac enlargement, rales, gallop rhythm, pulmonary venous congestion), or both, confirmed by noninvasive or invasive measurements demonstrating objective evidence of cardiac dysfunction	1
H	Hypertension	A resting blood pressure >140 mm Hg systolic and/or >90 mm Hg diastolic on at least two occasions or current antihypertensive pharmacological treatment	1
A_2_	Age, additional risk/point	Age ≥ 75 years	2
D	Diabetes	Fasting plasma glucose level ≥ 7.0 mmol/L (126 mg/dL) or treatment with hypoglycemic agent and/or insulin	1
S_2_	Thromboembolism	Either an ischemic stroke, transient ischemic attack, peripheral embolism, or pulmonary embolism	2
V	Vascular disease	Coronary artery disease (prior myocardial infarction, angina pectoris, percutaneous coronary intervention, or coronary artery bypass surgery) or peripheral vascular disease (the presence of any of the following: intermittent claudication, previous surgery or percutaneous intervention on the abdominal aorta or the lower extremity vessels, abdominal or thoracic vascular surgery, arterial and venous thrombosis)	1
A	Age standard risk/weight	Age = 65-74 years	1
Sc	Sex category	Female sex	1

Since it has been proven that all patients on anticoagulation possess a potential side effect of bleeding, in addition to the CHA2DS2-VASc score, prior to starting the anticoagulation regimen, the risk for bleeding ought to be assessed as well. This is achieved by means of the HAS-BLED scoring, which takes into account uncontrolled hypertension, abnormal renal or hepatic function, history of stroke, history of hemorrhages, labile international normalized ratio (INR), the age of the patient, and intake of drugs and other medications (Table 3) [18].

**Table 3:** Clinical risk factors in the HAS-BLED score. Reference [18]. SBP: systolic blood pressure; AST: aspartate aminotransferase; ALT: alanine aminotransferase; ALP: alkaline phosphatase; NSAID: non-steroidal anti-inflammatory drug; VKA: vitamin K antagonist; TTR: time in therapeutic range; INR: international normalized ratio.

Risk factors and definitions		Points awarded
H	Uncontrolled hypertension: SBP > 160 mmHg	1
A	Abnormal renal and/or hepatic function, dialysis, transplant, serum creatinine > 200 µmol/L, cirrhosis, bilirubin > x 2 upper limit of normal, AST/ALT/ALP > 3 x upper limit of normal	1 point for each
S	Stroke, previous ischemic or hemorrhagic stroke. (Hemorrhagic stroke would also score 1 point under the “B” criterion)	1
B	Bleeding history or predisposition. Previous major hemorrhage, anemia, or severe thrombocytopenia	1
L	Labile INR (only relevant is patient receiving a VKA), TTR < 60% in patients receiving VKA	1
E	Elderly age > 65 years in patients receiving VKA	1
D	Drugs or excessive alcohol drinking, concomitant use of antiplatelet or NSAIDs, and/or excessive alcohol per week. Alcohol excess or abuse refers to a high intake (e.g., >14 units per week), where the clinician assesses there would be an impact on health or bleeding risk.	1 point for each
Maximum score		9

Bleeding risk score comes with its own limitations, as it fails to assess the overall net clinical benefit of anticoagulation or make a comparison between the risk of bleeding and the risk of developing a stroke, and therefore cannot be used in isolation to prescribe DOACs. It has also been observed that some studies conclude the benefits of stroke prevention with oral anticoagulation generally outweigh the risks of bleeding, despite being at a high risk for bleeding [1]. Additionally, it is necessary to comprehend and take into consideration risk factors that are modifiable and unmodifiable. Non-modifiable risk factors include age greater than 65 years, prior medical history of severe hemorrhages, strokes, or small vessel disease, genetic factors such as a CYP2C9 polymorphism impairment, reduced functioning of the kidneys and liver, cancer, diabetes mellitus, or even dementia (Table 4) [18].

**Table 4:** Risk factors for bleeding with oral anticoagulants. Reference [18]. CYP: cytochrome P; CrCl: creatinine clearance; VKA: vitamin K antagonist; OAC: oral anticoagulant; INR: international normalized ratio; TTR: time in therapeutic range.

Non-modifiable	Potentially modifiable	Modifiable
Age more than 65 years	Extreme frailty and/or excessive risk of falls by appropriate footwear; home review to remove trip hazards; neurological assessment where appropriate and walking aids	Appropriate choice of OAC and correct dosing based on age, body weight, and serum creatinine levels
Past medical history of major hemorrhages, stroke, or small vessel disease	Anemia	Bridging therapy with heparin
Genetic factors (e.g., CYP 2C9 polymorphisms)	Reduced platelet count or function	INR control (target: 2.0-3.0), target TTR > 70% for patients receiving VKA treatment
Severe renal impairment (on dialysis or renal transplant) or hepatic dysfunction (cirrhosis)	Renal impairment with CrCl < 60 mL/min	Hazardous hobbies/occupations
Malignancy		Hypertension
Diabetes mellitus		Non-adherence to OAC
Cognitive impairment/dementia		Excessive alcohol intake

Anticoagulation therapies

Vitamin K Antagonists

Earlier, VKAs, like warfarin, were the only available option for stroke prevention in AF [19]. Warfarin inhibits an enzyme that activates vitamin K called “vitamin K epoxide reductase complex 1” (VKORC1). Since the synthesis of clotting factors II, VII, IX, and X and protein C and protein S (coagulation regulatory factors) are vitamin K-dependent, warfarin works by decreasing their production [20].

Though widely used, they remain far from ideal due to their narrow therapeutic intervals, varied monitoring requirements, and undesirable interactions with numerous drugs and foods [21,22]. The adverse effects of VKAs include bleeding in various parts of the body, for example, intracranial hemorrhage and GI bleeding [20]. Overall, the side effects of bleeding, irrespective of its severity, were more common in patients treated with VKAs than in NOAC-treated patients, antiplatelet-treated patients, and no-treatment patients [23].

Non-vitamin K Antagonist Oral Anticoagulants

Non-vitamin K antagonist oral anticoagulants have been slowly brought in over the last 10 years as an alternative to warfarin, as they addressed many of its drawbacks [24,25].

They work by competitively, selectively, and reversibly blocking either thrombin, called “direct thrombin inhibitors” (e.g., dabigatran), or factor Xa, called “direct factor Xa inhibitors” (e.g., apixaban, rivaroxaban, and edoxaban) [24,25]. Due to their fixed daily dosing, they are very convenient to the user. They have more stable anticoagulant effects with significantly lesser drug interactions and no stringent monitoring necessity [26].

Despite all this, they come with their fair share of disadvantages like higher acquisition costs, lack of international calibration standards for their coagulation assays, and lack of a system to deal with non-compliance. It has been established that 25-50% of all patients do not adhere to the prescriptions as directed by their doctors. In such scenarios, warfarin, with its long half-life (40 hours), can act as a “buffer” as opposed to the novel anticoagulants, which have shorter half-lives. Thus, non-adherence to these novel anticoagulants renders the patients more vulnerable to adverse effects [27].

Antiplatelet Drugs (APs)

They work by preventing clot formation by inhibiting different platelet receptors. Aspirin interferes with thromboxane A2 (TXA2) formation, while the P2Y12 adenosine diphosphate receptor is inhibited by clopidogrel and prasugrel, and dipyridamole blocks phosphodiesterase. The fact that they (aspirin mainly) are only useful in preventing very small non-cardioembolic strokes in comorbid patients with AF is possibly why they are not widely used for stroke prevention in AF [28].

Comparing the Efficacy of the Various Anticoagulants

A meta-analysis done with about 28,044 participants comparing the effectiveness of VKAs, antiplatelet therapy, and placebo concluded that VKA use in patients reduced the risk of stroke by 64% when put against placebo and by 39% when compared to single APs as long as the INR was maintained between 2.0 and 2.9 [2].

Comparing APs with warfarin and NOACs: Aspirin and P2Y12 inhibitors are antiplatelet drugs (APs), and were considerably less effective than oral anticoagulants in patients with AF, whether given alone or in combination [29]. So, it was indicated that overall, doses of warfarin that maintain an INR of around 2-3 are better in managing patients with AF than aspirin [27].

NOACs, when contrasted with VKAs, provide a 20% risk reduction in thromboembolic events, a 10% reduction in all-cause mortality, and a 50% drop in cerebral bleeds as evidenced by an individual patient data meta-analysis. Also, based on an observational study done in 2018, the largest of its time, between NOACs and warfarin, the NOACs reigned superior with reduced strokes and major bleeds in AF patients [30].

Current guidelines on stroke prevention in AF

The updated guidelines for the diagnosis and management of AF state that in patients with AF who possess an estimated annual thromboembolic risk of ≥ 2% per year (i.e., CHA2DS2-VASc score of ≥ 2 in men and ≥ 3 in women), anticoagulation is recommended to prevent stroke. For patients with AF who do not have a history of moderate to severe rheumatic mitral stenosis or a mechanical heart valve, and who are candidates for anticoagulation, DOACs are preferred to warfarin for stroke risk reduction. For AF patients with an estimated annual thromboembolic risk of ≥1% but <2% per year (equivalent to a CHA2DS2-VASc score of 1 in men and 2 in women), anticoagulation is reasonable to prevent stroke.

Currently, DOACs are the first line of therapy for stroke prevention in AF patients without mechanical heart valves or moderate to severe mitral stenosis. The DOACs, which are approved and prescribed at present, comprise dabigatran, a direct thrombin inhibitor, and three-factor Xa inhibitors, i.e., rivaroxaban, apixaban, and edoxaban (Table 5) [17,18].

**Table 5:** Dose selection criteria for NOACs. Reference [18]. NOACs: novel oral anticoagulants; b.i.d.: bis in die (twice a day); o.d.: omni die (once daily); CrCl: creatinine clearance.

Dose	Dabigatran	Rivaroxaban	Apixaban	Edoxaban
Standard dose	150 mg b.i.d.	20 mg o.d.	5 mg b.i.d.	60 mg o.d.
Lower dose	110 mg b.i.d.			
Reduced dose		15 mg o.d.	2.5 mg b.i.d.	30 mg o.d.
Dose-reduction criteria	Dabigatran 110 mg b.i.d. in patients with age ≥ 80 years, concomitant use of verapamil, or increased bleeding risk	CrCl = 15-49 mL/min	At least 2 of 3 criteria: age ≥ 80 years, body weight ≤ 60 kg, or serum creatinine ≥ 1.5 mg/dL (133 µmol/L)	If any of the following: CrCl = 15-50 mL/min, body weight ≤ 60 kg, or concomitant use of dronedarone, ciclosporin, erythromycin, or ketoconazole

Current guidelines for stroke prevention: DOACs versus warfarin

As per the 2023 ACC/AHA/American College of Clinical Pharmacy (ACCP)/Heart Rhythm Society (HRS) guidelines for the diagnosis and management of AF, direct oral anticoagulants are preferred over warfarin to prevent thromboembolic events. The only exception is patients with mitral stenosis or mechanical heart valves. Aspirin in either instance as monotherapy or in combination with clopidogrel as an alternative to anticoagulation is not recommended to reduce stroke risk. In patients with AF and chronic coronary artery disease (beyond one year after revascularization or coronary artery disease not requiring coronary revascularization) without a history of stent thrombosis, oral anticoagulant monotherapy is recommended over the combination therapy of oral anticoagulant and single antiplatelet agent (aspirin or P2Y12 inhibitor) to decrease the risk of major bleeding [31].

The 2023 ACC/AHA/ACCP/HRS guideline endorses the use of a validated clinical risk score, such as CHA2DS2-VASc, ATRIA (Anticoagulation and Risk Factors in Atrial Fibrillation), or GARFIELD-AF (Global Anticoagulant Registry in the FIELD-Atrial Fibrillation), to guide therapy in preventing thromboembolic events, and based on this estimation, clinicians are to make decisions (Table 6).

**Table 6:** Indications and contraindications for anticoagulants in patients with AF and associated cardiac comorbidities (European Society of Cardiology guidelines 2018). Reference [18]. AF: atrial fibrillation; DOAC: direct-acting oral anticoagulant.

Non-valvular AF	Contraindications
Intracardiac thrombus	Warfarin DOAC - increased risk of stroke or systemic embolism
Mechanical heart valve	Warfarin DOAC contraindicated
Moderate to severe mitral stenosis	Warfarin DOAC contraindicated
Other valve defects, mild-moderate	Warfarin DOAC
Severe aortic valve stenosis	Warfarin DOAC - limited data
Bioprosthetic valve (>3 months since implantation)	Warfarin DOAC - rivaroxaban is non-inferior to warfarin in bioprosthetic mitral valves, no robust data for the other DOACs, do not use DOAC in rheumatic etiology
Hypertrophic cardiomyopathy (HCM)	Warfarin DOAC - insufficient data, but may be considered
Transcatheter aortic valve implantation (TAVI)	Warfarin - no data for DOAC

Interventional procedures in stroke prevention

DOACs, also known as NOACs, have been the cornerstone for the prevention of stroke in patients with AF. Recent advancements in interventional approaches have been beneficial in preventing stroke in AF patients who either cannot take a DOAC due to contraindications, struggle with compliance, or suffer recurrent strokes despite anticoagulation. LAA is the most common site for the formation of blood clots and subsequent thrombo-embolization leading to stroke in non-valvular AF [32]. LAA occlusion or ablation forms the basis for the interventional approaches to prevent stroke.

Evolution of Interventional Procedures

Surgical appendectomy had been done in the 90s in patients undergoing concomitant cardiac surgeries. A randomized control trial on patients who underwent LAA appendectomy along with concomitant cardiac surgery for other indications had shown substantial risk reduction of stroke in AF [33]. It was also attributed that since cardiac diseases are a risk factor for developing AF, it was wise to perform an LAA appendectomy in the same setting.

**Figure 2:**
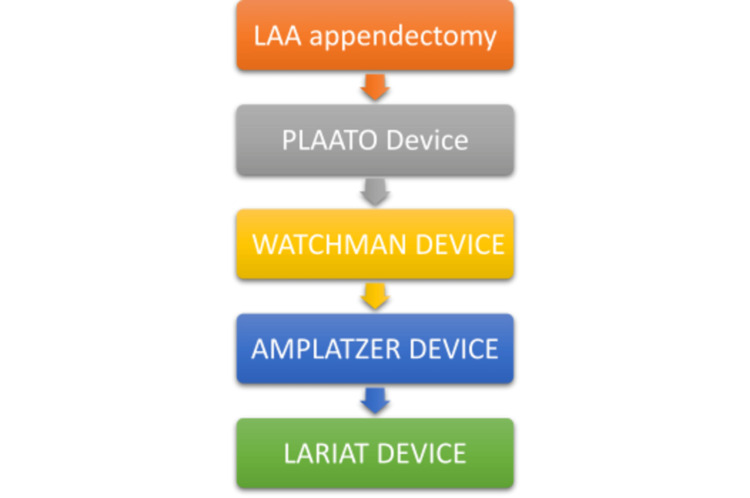
Timeline for the evolution of the interventional procedure. LAA: left atrial appendage; PLAATO: percutaneous left atrial appendage transcatheter occlusion.

Advancements in technology led to the development of percutaneous approaches for LAA occlusion in the early 2000s with devices such as PLAATO (percutaneous left atrial appendage transcatheter occlusion) [34]. The second dedicated LAA closure device that has been widely studied in randomized controlled trials is the WATCHMAN device [35]. The ASAP clinical trial has been instrumental in showing that the WATCHMAN device was successful in reducing the risk of thromboembolic stroke in AF patients who had a contraindication to oral anticoagulation therapy [36]. The study showed an ischemic stroke risk of 1.7% in patients who had WATCHMAN device implantation as compared to the expected risk of 7.3% based on CHADS2 scores of the patient cohort. The WATCHMAN device has been approved by Europe and USA. The other device called the AMPLATZER device has been shown to cause a 59% risk reduction in thromboembolism in a multicenter study involving 1047 patients [37]. The LARIAT study, in 2018, focuses on the epicardial LAA ligation using the LARIAT device, which has shown superior results to the WATCHMAN device [38]. It is also a percutaneous catheter-based LAA ligation with a suture delivery technique (Figure 2).

Interventional Procedures Versus DOACs

The European Society of Cardiology (ESC) guidelines have stated that oral anticoagulation should be considered first over LAA closure. Interventional procedures carry a risk of procedural complications, such as pericardial effusion, bleeding, ventricular perforation, and the risk of clot formation over the device. Furthermore, the majority of LAA occlusion procedures require antiplatelet therapy following the procedure, thus contributing to bleeding as with novel anticoagulants [39]. However, it is noteworthy that the bleeding risk and all-cause mortality rate with LAA occlusion have been observed to be significantly lower than the DOACs [40]. Hence, even with the passage of time, this risk remains low and offers benefits over the bleeding risks of DOACs. The study also observed that the risk of stroke in the left atrial appendage occlusion (LAAO) cohort was 2.1% per year and the risk in the DOAC cohort was 1.9%, which suggests that both therapies are almost equally efficacious (Table 7).

**Table 7:** DOACs versus interventional procedures. DOACs: direct-acting oral anticoagulants.

	DOACs	Interventional procedures
Risk of stroke post therapy	1.9%	2.1%
Bleeding risk	More	Less
Procedural complication risk	None	High
Concurrent antiplatelet therapy	Not required	Required post procedure
Discontinuation risk	High	None

Comparative analysis based on a recent network meta-analysis

Efficacy

In a network meta-analysis using individual patient-level data from the pivotal randomized trials of DOACs versus warfarin in patients with AF, Carnicelli et al. were able to draw conclusions regarding the efficacy and safety of the novel therapy [41]. DOACs demonstrated an overall higher efficacy in AF when compared to warfarin. It was noted that patients who received a standard-dose DOAC had a lower risk of stroke and systemic embolism (standard-dose DOAC: 883 out of 29,312 patients (3.01%) versus warfarin: 1080 patients out of 29,229 patients (3.69%); HR: 0.81, 95% CI: 0.74-0.89) over the duration of follow-up. Additionally, an overall reduction in the hazard of all-cause death and cardiovascular death was seen.

Patients who were randomized to a lower-dose DOAC regimen, which consists of either dabigatran 110 mg or edoxaban 30/15 mg, had no statistically different risk of stroke or systemic embolism (lower-dose DOAC: 531 patients out of 13,049 patients (4.07%) versus warfarin: 1080 patients out of 29,229 patients (3.69%); HR: 1.06, 95% CI: 0.95-1.19). However, yet again, in these patients, an overall reduction in the hazard of all-cause death and cardiovascular death was observed. In the case of a lowered dosage of DOAC, a higher risk of ischemic stroke was evident when compared with warfarin (lowered-dose DOAC: 454 patients out of 13,049 patients (3.48%) versus warfarin: 685 patients out of 29,229 patients (2.34%); HR: 1.35, 95% CI: 1.19-1.54) [41].

Safety

In terms of hazard of major bleeding, DOACs were shown to have no statistically different risk of major bleeding (DOACs: 1479 patients out of 29,270 patients (5.05%) versus warfarin: 1733 patients out of 29,187 patients (5.94%); HR: 0.86, 95% CI: 0.74-1.01). However, a decreased risk of fatal bleeding and intracranial bleeding has been proven, with the administration of DOACs, in comparison to warfarin.

An elevated risk for major gastrointestinal bleeding was observed in a standard dose regimen of DOACs (DOACs: 744 patients out of 29,270 patients (2.54%) versus warfarin: 569 patients out of 29,187 patients (1.95%); HR: 1.31, 95% CI: 1.08-1.57). However, after the sensitivity analysis, the estimated HR for major gastrointestinal bleeding with standard-dose DOAC versus warfarin was found to be lower in magnitude compared with the primary analyses. For standard-dose DOAC versus warfarin, the increase in major gastrointestinal bleeding observed in the primary analyses was no longer statistically significant (551/23,211 (2.37%) versus 436/23,189 (1.88%); HR: 1.24, 95% CI: 0.98-1.58). The risk of gastrointestinal bleeding with standard-dose DOACs is counterbalanced by drastic reductions in intracranial bleeding, thromboembolism, and massive hemorrhages, which are more significantly alarming outcomes (Table 8).

**Table 8:** Comparative analysis of meta-analysis. DOACs: direct-acting oral anticoagulants.

	Traditional anticoagulants	DOACs	Inference
Risk of stroke and systemic embolism	Warfarin: 1080 patients out of 29,229 patients (3.69%)	Standard dose: 883 patients out of 29,312 patients (3.01%)	Lowered risk in standard-dose DOACs
	Warfarin: 1080 patients out of 29,229 patients (3.69%)	Lowered dose: 531 patients out of 13,049 patients (4.07%)	No statistically different risk in lowered-dose DOACs
Risk of ischemic stroke	Warfarin: 685 patients out of 29,229 patients (2.34%)	Lowered dose: 454 patients out of 13,049 patients (3.48%)	Higher risk in lowered-dose DOACs
Risk of major bleeding	Warfarin: 1733 patients out of 29,187 patients (5.94%)	Standard dose: 1479 patients out of 29,270 patients (5.05%)	No statistically different risk in standard-dose DOACs
	Warfarin: 1733 patients out of 29,187 patients (5.94%)	Lowered dose: 564 patients out of 12,985 patients (4.34%)	Lowered risk in lowered-dose DOACs
Risk of major gastrointestinal bleeding	Warfarin: 436 patients out of 23,189 patients (1.88%)	Standard dose: 551 patients out of 23,211 patients (2.54%)	No statistically different risk in standard-dose DOACs
	Warfarin: 569 patients out of 29,187 patients (1.95%)	Lowered dose: 271 patients out of 12,985 patients (2.09%)	No statistically different risk in lowered-dose DOACs

In patients who received a lower dose of DOAC, a reduced risk of major bleeding was seen (DOACs: 564 patients out of 12,985 patients (4.34%) versus warfarin: 1733 patients out of 29,187 patients (5.94%); HR: 0.63, 95% CI: 0.45-0.88). Lowered incidences of clinically relevant nonmajor bleeding, intracranial bleeding, and fatal bleeding were also inferred. The same patients who received a lower-dose DOAC showed no statistical difference in the risk of major gastrointestinal intestinal bleeding (DOACs: 271 out of 12,985 patients (2.09%) versus warfarin: 569 out of 29,187 patients (1.95%); HR: 0.85, 95% CI: 0.62-1.18).

It is worth noting that the beneficial outcome of the standard dosing regimen of DOACs is more clinically evident when a history of VKA use is absent and in patients with lower creatinine clearance. A higher benefit for standard dosing of DOACs is distinguishable in AF patients with lower body weight and younger age, irrespective of sex [41].

When evaluating the efficacy of drugs in stroke prevention, warfarin is found to be quite effective in reducing stroke incidence by 64%. However, in comparison to NOACs, it was found that all NOACs were superior to warfarin in stroke prevention. At the same time, NOACs claimed to have a slightly better safety profile in terms of mortality rate and major or intracranial bleeding than traditional anticoagulants (Table 9).

**Table 9:** Comparative analysis of various clinical trials. NOACs: novel oral anticoagulants; DOAC: direct-acting oral anticoagulant.

Parameters	Traditional anticoagulants	NOACs currently in use
Efficacy: stroke reduction [42]	Warfarin: 64% (Robert G Hart). Overall reduction in stroke prevention	∙ Dabigatran: 34% (RE-LY trial) ∙ Rivaroxaban: 12% (ROCKET AF trial) ∙ Apixaban: 21% (ARISTOTLE trial) ∙ Edoxaban: 21% (ENGAGE AF-TIMI 48 trial). Reduction in stroke prevention compared to warfarin
Safety, mortality rate [43-46]	Warfarin: 4.13% (RE-LY trial)	∙ Dabigatran: 3.64% vs. 4.1% (RE-LY trial) ∙ Rivaroxaban: 3.2% vs. 3.4% (ROCKET AF trial) ∙ Apixaban: 3.5% vs. 3.9% (ARISTOTLE trial) ∙ Edoxaban: 2.7% vs. 3.4% (ENGAGE AF-TIMI 48 trial). DOAC vs. warfarin
Side effects: intracranial bleeding [43-46]	Warfarin: 0.74% (RE-LY trial)	∙ Dabigatran: 0.3% vs. 0.74% (RE-LY trial) ∙ Rivaroxaban: 0.5% vs. 0.7% (ROCKET AF trial) ∙ Apixaban: 0.33% vs. 0.8% (ARISTOTLE trial) ∙ Edoxaban: 0.5% vs. 0.8% (ENGAGE AF-TIMI 48 trial). DOAC vs. warfarin
Side effects: major bleeding [43-46]	Warfarin: 3.36% (RE-LY trial)	∙ Dabigatran: 3.11% vs. 3.36% (RE-LY trial) ∙ Rivaroxaban: 2.7% to 3.6% vs. 2.1% to 3.4% (ROCKET AF trial) ∙ Apixaban: 2.2% vs. 3.1% (ARISTOTLE trial) ∙ Edoxaban: 2.8% vs. 3.4% (ENGAGE AF-TIMI 48 trial). DOAC vs. warfarin
Patient adherence at one year [47]	Warfarin: 37.2%	∙ Dabigatran: 68% ∙ Rivaroxaban: 67% ∙ Apixaban: 70% ∙ Edoxaban: 77%
Cost-effectiveness [48]	Warfarin: 9.02	∙ Dabigatran: 9.35 ∙ Rivaroxaban: 9.24 ∙ Apixaban: 9.38 ∙ Edoxaban: 9.31
Reversal agent [49]	Warfarin: vitamin K, prothrombin complex, and fresh frozen plasma	∙ Dabigatran: Idarucizumab ∙ Rivaroxaban: Andexanet alfa ∙ Apixaban: Andexanet alfa ∙ Edoxaban: Andexanet alfa

These differences in the safety profile of the two drug classes also translate to the patient adherence rate as patients are significantly more inclined to continue dosing with NOACs ~70% at the end of one year compared to 37% for warfarin. NOACs were also found to be more cost-effective.

Notably, all these drugs have potent reversal agents in the market, but there are multiple options for reversing the action of warfarin. Also, these are cheaper and much more easily attainable than the reversal agents of NOACs, such as idarucizumab, which is an antibody targeting dabigatran or recombinant factor Xa, and andexanet alfa, which counteracts the effects of other novel anticoagulants.

## Conclusions

NOACs, including dabigatran, rivaroxaban, apixaban, and edoxaban, have significantly transformed stroke prevention in patients with AF. Unlike VKAs like warfarin, NOACs do not require routine monitoring, improving patient adherence. They are preferred over warfarin, aspirin, and other antiplatelet drugs, which have limited efficacy in stroke prevention for AF patients. NOACs have demonstrated better safety profiles, showing lower risks of major and intracranial bleeding, and a 20% reduction in thromboembolic events, along with a 10% reduction in overall mortality compared to VKAs.

Effective anticoagulation therapy is essential for stroke prevention in AF patients due to their high risk of thromboembolism. Current guidelines recommend oral anticoagulation unless the estimated annual thromboembolic risk is below 2%, assessed using the CHA2DS2-VASc score, while bleeding risk is evaluated using the HAS-BLED score. Maintaining proper anticoagulation reduces stroke incidence, improves survival rates, and enhances patients' quality of life. Additionally, interventional procedures like LAA occlusion offer alternatives for patients contraindicated for anticoagulation therapy. A patient-centered approach considering risk factors, co-morbidities, and personal preferences is crucial in guiding the choice of anticoagulant therapy.
